# In the Spotlight—Established Researcher

**DOI:** 10.1002/jez.b.23166

**Published:** 2022-06-08

**Authors:** Abderrahman Khila

**Affiliations:** ^1^ Institut de Génomique Fonctionnelle de Lyon, Centre National de la Recherche Scientifique Unité Mixte de Recherche 5242, Ecole Normale Supérieure de Lyon, Université de Lyon Université Claude Bernard Lyon 1 Lyon France


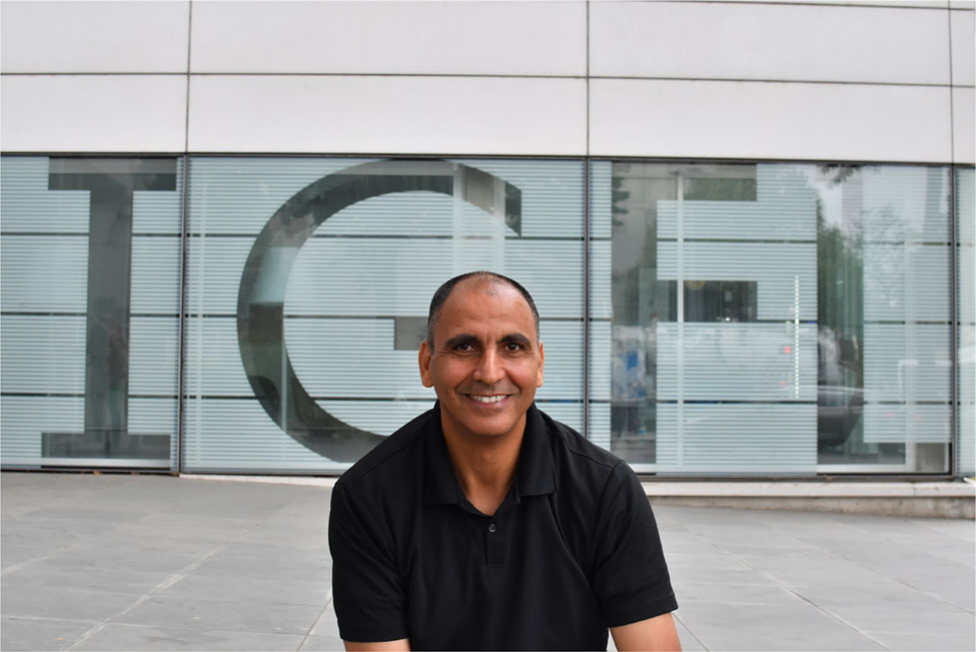


Abderrahman Khila is a recipient of ATIP‐Avenir (CNRS) and ERC Consolidator (Europe), Fondation Recherche Médicale, Agence Nationale de la Recherche grants. He is an Academic Editor at *PLoS Biology*, and an Associate Editor at *Evolution Letters*, EvoDevo and former Associate Editor at *BMC Evolutionary Biology*. Khila is a guest professor at the department of Ecology and Genetics, Evolutionary Biology, Uppsala University, Sweden.

Website: https://igfl.ens-lyon.fr/equipes/a.-khila-developmental-genomics-and-evolution

Pubmed: https://pubmed.ncbi.nlm.nih.gov/?term=Khila%2Ba

Google Scholar: https://scholar.google.com/citations?user=fUuLtAkAAAAJ

1

***With whom and where did you study?***

My career started with a Master's degree in Nutrition and Food Sciences in Morocco, although my main interest already back then was in genetics. I did a joint PhD in Molecular, Cell and Developmental Biology between the University of Fez in Morocco and the University of Toulouse in France. I was advised from the Moroccan side by Dr. Saad Ibnsouda and the French side by Dr. Alain Vincent. I also had the great privilege to be mentored by Dr. François Payre. The aim of the PhD project was to reproduce the ovoD1 female sterile mutation in the olive fruit fly and establish a genetically controlled sterile insect technique for pest management.

2

***What got you interested in biology? When did you know EvoDevo was for you?***

Biology has always been a natural choice for me, primarily because I loved nature since my childhood, but also because I grew up with older siblings who were excited about biology. Having grown up during the 80s in a small town in the South East of Morocco called Ouarzazate, we enjoyed a degree of freedom from our parents that I cannot even think of allowing myself today. I spent most of my free time with friends along a seasonal river fishing, exploring, and often getting into trouble with local farmers. At school, I had a natural leaning toward the natural sciences, and I feel extremely lucky to have had teachers at various levels, who deeply reinforced my interest in Biology.

I discovered EvoDevo superficially during my PhD and then became part of the community during my first postdoctoral position at the Western University in Ontario, Canada. But my real excitement about EvoDevo started at McGill University, Canada, when I joined the lab of Ehab Abouheif to work on ant development and social evolution. This was truly an experience that allowed me to move from biotechnology‐driven projects to fundamental discovery‐driven thinking. This experience expanded when Ehab and I teamed up with Locke Rowe from the University of Toronto to include projects dealing with sexual conflict and water surface locomotion in water striders. This was something I enjoyed doing because it allowed me to connect the power of developmental genetics with important evolutionary concepts, such as sexual selection and social evolution.

3

***Who was your most influential mentor?***

Many, it is difficult to pick one as I learned different things at different stages from different people. My adviser in Morocco, Dr. Saad Ibn Souda, took a chance on me and taught me the first basics of Molecular and Developmental Biology. I would not be here if he had not opened the door for me. Dr. Alain Vincent saw potential in me and opened his lab when he offered me a joint PhD position in Toulouse. François Payre was my day‐to‐day mentor in Toulouse and taught me many things from science to life in general. With Ehab Abouheif at McGill, I had the chance to mature as a PI and I learned from him how to write papers and grants and how to transform ideas into real scientific projects. I also learned a great deal of Evolutionary Biology and sexual selection concepts from Locke Rowe. Many others contributed, and some just out of their desire to support a younger colleague, and here I am thinking about Claude Desplan from NYU in particular.

4

***Which achievement are you most proud of?***

Having had a significant contribution to establishing a full lineage (water striders or Gerromorpha) as a model is something that I hope the field will remember and that the foundation I am building will serve long into the future. For this, I want to thank all my team members, past and present, and my collaborators. This amazing system allowed us to bridge multiple disciplines, from genomics and genetics to ecology, evolution, and even biomechanics. Some of the works from my group, led by Emila Santos now an assistant professor at the University of Cambridge UK, that I hope will become a classic, is a study showing how new genes can drive novelties which in turn help lineages access new ecological opportunities. During my postdoctoral time, Ehab Abouheif and I published a paper where we applied developmental biology concepts to ask questions about reproduction and social organization in ants. This study removed an important argument whereby workers can produce males due to haplodiploidy and therefore create a conflict over reproduction in the colony. We heard both praise and criticism for this study, which somehow felt good.

5

***What do you see as the future of EvoDevo?***

EvoDevo is a fast‐changing discipline that contributes to and benefits from the impressive advances in technologies such as imaging, mass sequencing, and single‐cell data acquisition and analyses. A number of questions in EvoDevo have suddenly become accessible thanks to these technologies and the field is poised to benefit. However, EvoDevo will also benefit from a better focus on the concepts and questions related to phenotypic evolution, especially in concert with other sister disciplines such as Ecology and Evolution, and population and quantitative genetics. Bridges must be built to other disciplines as many of the answers sought are shared across fields. A better incorporation of fieldwork into EvoDevo would also be an asset.

